# Green Space and Depression during Pregnancy: Results from the *Growing Up in New Zealand* Study

**DOI:** 10.3390/ijerph14091083

**Published:** 2017-09-18

**Authors:** Vikram Nichani, Kim Dirks, Bruce Burns, Amy Bird, Cameron Grant

**Affiliations:** 1 Section of Epidemiology and Statistics, School of Population Health, University of Auckland, Auckland 1142, New Zealand; k.dirks@auckland.ac.nz; 2 School of Biological Sciences, University of Auckland, Auckland 1142, New Zealand; b.burns@auckland.ac.nz; 3 Centre for Longitudinal Research he Ara ki Mua, School of Population Health, University of Auckland, Auckland 1142, New Zealand; a.bird@auckland.ac.nz (A.B.); cc.grant@auckland.ac.nz (C.G.); 4 Department of Pediatrics: Child and Youth Health, School of Medicine, University of Auckland, Auckland 1142, New Zealand; 5 General Pediatrics, Starship Children’s Hospital, Auckland District Health Board, Auckland 1023, New Zealand

**Keywords:** antenatal depression, green spaces, census area units, geographic information systems, multilevel data

## Abstract

*Background*: Antenatal depression is an important contributor to poor maternal health experienced by some women. This study aimed to determine whether exposure to green space during pregnancy is associated with less depression, and whether this association is moderated by relevant factors, such as age, education, self-identified ethnicity, physical activity, residential rurality, and socioeconomic status. *Methods*: Health data were sourced from the cohort study “*Growing Up in New Zealand*” comprised of 6772 participants. Green space was estimated based on the proportion of green space within the Census Area Unit. Adjusted logistic mixed effect models were used to investigate the association between green space and antenatal depression after controlling for confounding variables. *Results*: Maternal exposure to green space were not associated with lower odds of antenatal depression. Indications of effect modifications due to relevant factors were not observed. *Conclusions*: This study did not determine an association between access to green space (measured based on the distance to the nearest green space) and antenatal depression. Therefore, a link between green space and antenatal depression was not established. For that reason, ensuring residential areas contain adequate green space may or may not be helpful in preventing antenatal depression and adverse health outcomes associated with this depression. More studies focusing on pregnant women in a range of social contexts, and considering both exposure and access to green space, are warranted to determine the relationships between green space and antenatal depression.

## 1. Introduction

According to the World Health Organization, the most common mental health disorder affecting adults in the general population is depression [1]. In the United States of America (USA), billions of dollars each year are spent on depression, attributable to direct medical costs (i.e., medical services and prescription drug costs), suicide-related mortality, and workplace costs (i.e., costs associated with an absence from work and reduced productivity) [2]. Depression is clinically diagnosed by the presence of unhappiness; feelings of guilt; tiredness; and lack of appetite, sleep, concentration, and pleasure [1]. The prevalence rate of depression amongst adults in the general population is country-specific, ranging from 11% for low and medium income countries to 15% for high income countries [3]. Depression can manifest as a chronic condition, and, if left untreated, leads to loss of productive life years during the life course of the affected individual [1,4]. In 2001, depression was regarded as the fourth leading cause of disability [5]. By 2020, depression is projected to become the second most important factor associated with disability [5]. In the most severe cases, depression culminates into suicide [1].

Globally, variable prevalence rates of antenatal depression (defined as “depression during pregnancy”) have been observed, ranging from 12% to 20% of pregnant women [6]. In New Zealand, it is estimated that 15% of pregnant women (hereafter referred to as women) suffer from mental disorders, including anxiety and depression [7]. Antenatal depression is an important contributor to the adverse pregnancy outcomes of low birth weight, preterm birth, small for gestational age, smaller head infants, and adverse child health outcomes of low Apgar scores and infant mortality [8,9]. Additionally, antenatal depression is one of the important etiological factors responsible for the development of postnatal depression [10,11]. Postnatal depression in mothers is linked to higher cognitive, behavior, and interpersonal problems in their children [12]. 

One environmental intervention that can combat antenatal depression in women is exposure to green space [13]. The United States Environmental Protection Agency has defined green space as “land that is partly or completely covered with grass, trees, shrubs, or other vegetation” [14]. It has been observed that women living in space that is more green are less likely to develop antenatal depression [13]. At the same time, exposure to green space increases the levels of physical activity in women [13] and children [15]. In this context, recent studies have indicated that the development of large green space areas are frequently preferred in landscape planning stages [16,17,18]. The beneficial effects of green space exposure during pregnancy are more evident in women of lower socioeconomic status [13], those with low levels of education [19,20,21,22], or in those living in highly deprived areas [21]. More specifically, recent studies on green space and pregnancy outcomes indicate that women with low or medium levels of education deliver higher birth weight infants compared with women with high levels of education [19,20,21,22]. The association of green space exposure with higher birth weight is stronger among women residing in the most deprived areas compared with women residing in the moderate or least deprived areas [21]. Thus, a moderating effect of socioeconomic status in the association between green space and pregnancy outcomes has been demonstrated. At the same time, data from some general population studies are suggestive of a moderating effect of age in the association between green space and mental health [23,24], and a moderating effect of residential rurality in the association between green space and general health [25]. More exactly, general population studies on the associations between exposure to green space and mental health outcomes have demonstrated that green space is beneficial for the mental health of people within specific age groups (e.g., 18–24, <30, and 31–50 years) [23,24]. A study of the association between exposure to green space and health outcomes amongst general population adults has shown that people living in greener areas have better health outcomes (e.g., morbidity symptoms and perceived general health status) in comparison with those living in low-green areas; these associations are seen mainly in people living in the slightly urban/moderately urban/nonurban areas [25]. In the general population, the association between green space with mental health outcome of psychological distress is more prominent in people who are physically active [26]. That is, people who live in areas of high green space and who are physically active are less likely to develop psychological distress in comparison with people who live in low green areas and who are physically inactive [26]. It is also known that green space is associated with better mental health in women, at least in part through increased participation in physical activity [13]. One limitation that appears in previous general population studies [27,28], and those focused specifically on women [13] and green space in relation to mental health, is that the studies have not accounted for nor controlled for self-selection bias in the regression analyses. 

We aimed to investigate whether exposure to green space for women was associated with a lower likelihood of antenatal depression after accounting for confounders, including socioeconomic status and the length of stay at their current residence, used as a surrogate for self-selection bias. We also aimed to investigate whether the effect of green space exposure on antenatal depression varied between different age and ethnic groups, low/medium/high levels of education, urban/rural groups, low/medium/high area deprivation groups, and for physically active groups. We sourced health data from a cohort that sampled women of diverse ethnicity and socioeconomic status, and gathered data on physical activity, so that the effect of modifications of demographic and residential factors and physical activity on the relationship between green space exposure and antenatal depression could be investigated.

## 2. Materials and Methods

### 2.1. Study Source

Data for this study were sourced from mothers who were participants in the *Growing Up in New Zealand* study, a longitudinal pre-birth cohort study of 6853 children and their parents who are residents of the Auckland, Manukau, or Waikato regions of New Zealand [29]. The study region, covered by three adjacent District Health Boards of Auckland, Counties Manukau and Waikato, represented 11% of the live births in New Zealand, from March 2009 to May 2010 [29]. *Growing Up in New Zealand* recruited 6822 women for the first data collection wave, called the “antenatal wave”, whereby data were collected through face-to-face interviews with women [29]. Participants of the *Growing Up in New Zealand* study were interviewed prior to the birth of their child or children, as well as after the birth [29]. Written informed consent was obtained from mothers for their participation, as well as of their unborn children [29]. As part of the antenatal wave, data that described demographics, health behaviors and history, and household characteristics were collected from mothers [29].

### 2.2. Estimation of Exposure to Green Spaces

The assessment of green space was performed based on the proportion of green space within a given census area unit (CAU). CAUs in New Zealand are the second smallest geographical units consisting of populations of 3000 to 5000 [30]. Statistics New Zealand define census area units as “non-administrative areas that are in between mesh blocks and territorial authorities in size” [30]. Our method of assessment of green space was similar to that used in previous studies in New Zealand [31,32,33], namely, by dividing green spaces within CAUs into different quartiles based on the percentage of green space in the CAUs. Our definition of green space included green areas, such as parks, beaches, urban parklands/open spaces, forests, grasslands, and croplands, but excluded private gardens. Other non-green areas (e.g., built-up areas (e.g., commercial, industrial, and residential buildings), space used to support transport infrastructure (e.g., roads, rail-yards, and airport runways) and water bodies (e.g., rivers and lakes)) were also excluded from our measure of green space. Data on green space for the study region were sourced from the Auckland Council [34] and the Waikato District Council. We supplemented data on green space from two Councils with data on green space from the New Zealand Land Cover Database (LCDB) of the Land Resource Information Systems portal [35]. The procedure of combining data on green space provided more attributes than using data from a single source. Green space data from the Waikato District Council had a scale of 1:50,000 and an accuracy of 90.0%. The LCDB data had a scale of 1:50,000 and an accuracy of 93.9% [35]. As the relationship between green space and antenatal depression was non-linear, the green space variable was utilized as a categorical variable. We took the 25th, 50th, and 75th percentiles as the break points for the categorization of green space. The utilization of those percentiles resulted in green space being categorized in our study as low (0% to <12%), medium (12% to <21%), high (21% to <38%), and very high (38% to 100%). The Aeronautical Reconnaissance Coverage Geographic Information System (ArcGIS) Version 10.3 (Environmental Systems Research Institute, Redlands, CA, USA) was used to perform the green space analyses. 

### 2.3. Covariates

The covariates used were age (categorized as <20, 20–24, 25–29, 30–34, 35–39, and ≥40 years), ethnicity (defined as self-identified ethnicity and categorized into European, Māori (New Zealand’s indigenous population), Pacific, Asian, Middle Eastern/Latin-American/African, and New Zealander/Other), educational attainment (defined as the highest level of education attained and categorized as no secondary school qualification, secondary school, diploma certificate, bachelor’s degree, and higher degree), employment status (defined as status in labor force service and categorized as employed, unemployed, student, and not in work force), area deprivation (defined as “the New Zealand Deprivation Index 2006 [NZDep2006]”) which is obtained by combining a set of variables collected during the 2006 national census (e.g., income, home ownership, living space, access to telephone, and access to car) [36], and categorized into deprivation deciles of low (deciles [1,2,3]), medium (deciles [4,5,6,7]), and high (deciles [8,9,10]), smoking status (defined as smoking of cigarettes during pregnancy, and categorized as yes or no), alcohol consumption (defined as consumption of alcohol during pregnancy, and categorized as no drinking during pregnancy or any drinking during pregnancy), relationship status with biological father (defined as social relationship status with biological father, and categorized as no relationship; dating, not cohabiting; cohabiting; and married or civil union), parity (defined as the number of pregnancies and categorized as first or subsequent), residential rurality (defined as residence in urban or rural areas), physical activity during and after the first trimester of pregnancy (defined as participation in recommended levels of physical activity of at least 150 min per week [37], and categorized as yes or no), pre-pregnancy general health status (defined as general health status during pre-pregnancy period and categorized as poor/fair, good, very good, or excellent), and the length of stay at the current residence (measured in years and described below) [29].

### 2.4. Self-Selection Bias

The importance of self-selection bias has been recognized in studies on green space and health. For example, a systematic review of the association between measures of the built environment (e.g., parks and public open spaces) and physical activity amongst adults from the general population has identified that neighborhood self-selection is likely to be a confounder of the association between measures of the built environment and physical activity [38]. One way to reduce the creation of biased estimates while determining the association between measures of the built environment and physical activity is to statistically control for neighborhood self-selection [39].

It is likely that the association between exposure to green spaces and depression amongst general population adults is confounded by the process of neighborhood self-selection [40]. This choice could, at least in part, explain an association between exposure to green space and depression amongst general population adults [40]. Consequently, the length of stay at the current residence had to be considered while examining the association between green space and antenatal depression in this study.

Consistent with previous research studies on exposure to green space and mental health outcomes amongst general population adults [40,41], we used the variable “length of stay at current residence” as a surrogate measure for neighborhood self-selection. This is taken into account because a minimum length of time (at least one year of stay at the current residence [42]) is needed before the beneficial effects of exposure to green space on mental health become evident. In the *Growing Up in New Zealand* study, the variable “length of stay at current residence” was described as the number of years that the women had lived in their current residence [43], framed as “How long have you lived in this current home?” and specifying the number of months, or number of years, or both, that they lived in their current home [43].

### 2.5. Dependent Variable

The dependent variable utilized in this study was the Edinburgh Postnatal Depression Scale (EPDS) as it is the most common screening instrument used for the detection of antenatal and post-natal depression [44,45]. The EPDS questionnaire consists of 10 questions that extract in-depth information on antenatal or postnatal depression [44,45]. Each question has four responses (e.g., Yes, most of the time; Yes, quite often; Not very often; and No, not at all) and a rating score of 0–3 points, with the maximum calculated total score for any individual being 30 points [46]. Both the validity and reliability of the EPDS have been demonstrated for its usage in diverse cultures [47,48,49]. For women who had limited ability to speak English, or those who could not speak the English language, an interpreter was available so that the EPDS questionnaires could be administered.

In the *Growing Up in New Zealand* study, women were asked to recollect information over the past seven days while answering the following ten questions of the EPDS questionnaire: (1) I have been able to laugh and see the funny side of things, (2) I have looked forward with enjoyment to things, (3) I have blamed myself unnecessarily when things went wrong, (4) I have been anxious or worried for no good reason, (5) I have felt scared or panicky for no very good reason, (6) Things have been getting on top of me, (7) I have been so unhappy that I have had difficulty sleeping, (8) I have felt sad or miserable, (9) I have been so unhappy that I have been crying, and (10) The thought of harming myself has occurred to me. After gathering information from the EPDS questionnaires, the categorizations of antenatal depression and non-depression in the *Growing Up in New Zealand* study were made at a cut-off value of a total score of 13 points. At the threshold value of 13 points, the sensitivity for antenatal depression is 0.83 and specificity for antenatal depression is 0.90 [46]. A total score of ≥13 points was considered to be associated with a high risk for antenatal depression [43]. For the analyses relating to the current study, the EPDS scores for women were dichotomized as 0 (absence of antenatal depression) or 1 (presence of antenatal depression) using the same cut-off value of 13 points.

### 2.6. Statistical Analyses

The final sample of 6772 mothers in this study from the original cohort of 6822 mothers was created by restricting analyses to those women for whom each geocoded CAU number was available for analysis. There were 613 CAUs within the study regions. On average, 11 respondents resided in each CAU. If the clustering of respondents in CAUs of study regions is not accounted for within the regression models, the standard errors of the contextual effect estimates are likely to be downwardly biased. Multilevel designs are ideally suited to the analysis of neighborhood effects, such as green space, within different sized CAUs, by simultaneously analyzing individual and neighborhood-level variables, whilst accounting for the non-independence in the data [50]. As the proportion of missing values in the variables was low, we decided to perform a complete case analysis. We conducted a multilevel mixed model for a green space-depression association using district health board of the maternal domicile as a random effect. As the EPDS score could not be normalized, we used EPDS as a binary variable. The length of stay at the current residence was modeled as a continuous variable and the green space-depression association judged based on logistic regression by observing odds ratios (ORs) and their 95% confidence intervals (CIs). Firstly, we conducted univariate regression analyses (Null Model) to determine whether exposure to green space by itself was a significant predictor of antenatal depression. Subsequently, confounders were added to the null models one by one to determine whether exposure to green space remained a significant predictor of antenatal depression. Interaction tests determined the possibility of interactions between green space and relevant variables. The final regression model had all independent variables, including green space, and interaction terms if they led to a better fit in the likelihood ratio tests. A total of seven mixed models were developed in Stata Version 14 (Stata Corporation, College Station, TX, USA) for investigating the association between green space and antenatal depression. 

## 3. Results

### 3.1. Exposure to Green Spaces

The median area within CAUs of the Auckland and Counties Manukau District Health Board regions was 1.62 km^2^ (*n* = 413 CAUs). On the other hand, the median area within CAUs of the Waikato District Health Board region was 6.64 km^2^ (*n* = 200 CAUs). The mean (standard deviation (SD)) percentage of green space in the CAUs for the Auckland and Counties Manukau District Health Board regions was 38% (32%). Correspondingly, the mean (SD) percentage of green space in the CAUs of the Waikato District Health Board region was 65% (34%). 

Educational attainment (*χ*^2^ = 47.01; *p* < 0.0001) and self-identified ethnicity (*χ*^2^ = 352.00; *p* < 0.0001) were significantly different among population subgroups in terms of their exposure to green space. Women who had acquired diploma level education qualifications were exposed to higher surrounding greenness within CAUs of their residence than those with other levels of educational qualification (diploma = 32.65% (95% CI = 31.42–33.88%), no secondary school = 31.05% (95% CI = 28.70–33.40%), secondary school = 30.83% (95% CI = 29.50–32.16%), bachelor’s degrees = 30.63% (95% CI = 29.19–32.07%), and higher degrees = 28.00% (95% CI = 26.38–29.63%)). European women were exposed to higher surrounding greenness within CAUs of their residence than non-European women (European = 36.14% (95% CI = 35.10–37.17%), Māori = 31.08% (95% CI = 29.43–32.73%), Pacific = 21.90% (95% CI = 20.93–22.86%), Asian = 21.31% (95% CI = 20.14–22.50%), Middle Eastern/Latin-American/African = 26.58% (95% CI = 23.12–30.05%), and Other or New Zealander = 34.95% (95% CI = 28.42–41.48%)).

Exposure to green space was also significantly different for population subgroups based on the level of area deprivation (F = 172.79; *p* < 0.001). Mothers residing in low deprivation areas were exposed to higher levels of green space in comparison to those residing in medium and high deprivation areas. More specifically, the proportion of green space available to women residing in low, medium, and high deprivation areas was 39.93% (95% CI = 38.33–41.53), 31.85% (95% CI = 30.68–33.02), and 24.23% (95% CI = 23.50–24.96), respectively. 

### 3.2. Descriptive Statistics and Bivariate Analyses

The mean (SD) of the maternal age of mothers enrolled in the *Growing Up in New Zealand* study was 30 (6) years, with most of these women, 93%, residing in urban areas at the time of cohort enrollment (Table 1). The mean (SD) duration of the length of stay at the current residence for mothers was 4 (6) years (Table 1). Fifteen per cent of these mothers experienced depression during the antenatal period, and the proportion of the study cohort experiencing antenatal depression who resided in areas of low, medium, high, and very high green space were 14.68%, 17.32%, 17.45%, and 15.22%, respectively.

Significant differences were identified among population subgroups defined by NZDep2006 in terms of length of stay at the current residence (F = 1.62; *p* < 0.0001). The mean lengths of stay at the current residence for women residing in low, medium, and high deprivation areas were 3.89, 3.96, and 4.86 years, respectively. In contrast, the length of stay at the current residence was not associated with green space (F = 1.15; *p* = 0.10), residential rurality (F = 0.95; *p* = 0.68), physical activity during (F = 1.15; *p* = 0.09) and after the first trimester of pregnancy (F = 0.93; *p* = 0.74), and antenatal depression (F = 1.00; *p* = 0.50).

### 3.3. Interaction Tests for the Association of Green Space with Antenatal Depression

We did not find any interactions between green space and relevant factors while determining the association between green space and antenatal depression (area deprivation (*p* = 0.07), physical activity during the first trimester of pregnancy (*p* = 0.07), age (*p* = 0.09), physical activity after the first trimester of pregnancy (*p* = 0.45), residential rurality (*p* = 0.75), self-identified ethnicity (*p* = 0.83), and educational attainment (*p* = 0.94). We did not perform any subgroup analyses for groups of physical activity after the first trimester of pregnancy, residential rurality, self-identified ethnicity, and educational attainment. The *p*-values for interactions between green space and relevant factors, such as area deprivation, physical activity during the first trimester of pregnancy, and age, were close to significance; therefore, we performed subgroup analyses for different areas of deprivation, physical activity during the first trimester of pregnancy, and age groups. The results of these subgroup analyses were similar to those of the main analyses, and indicative of lacking associations between green space and antenatal depression (data not shown).

### 3.4. Main Analyses for the Association of Green Space with Antenatal Depression for the Entire Cohort

Univariate logistic regression analysis revealed that exposure to medium, high, or very high levels of green space was not associated with antenatal depression (unadjusted OR medium green space = 1.11 (95% CI = 0.91–1.36); unadjusted OR high green space = 1.12 (95% CI = 0.92–1.36); and unadjusted OR very high green space = 0.95 (95% CI = 0.77–1.17)) (Null Model). Similarly, no association was found between exposure to green space and antenatal depression after accounting for all confounders. That is, the fully adjusted multivariate regression analyses (Model 7) showed that exposure to medium, high, or very high levels of green space was not associated with antenatal depression (adjusted OR medium green space = 1.10 (95% CI = 0.89–1.35); adjusted OR high green space = 1.15 (95% CI = 0.94–1.41); and adjusted OR very high green space = 1.21 (95% CI = 0.96–1.52)) (Table 2).

## 4. Discussion

### 4.1. Main Findings

The range of green space exposure for the participants in this study was similar to the range of green space exposure found in previous studies conducted in New Zealand [31,32,33]. Additionally, in this study, we observed a socioeconomic gradient in exposure to green space. That is, green space decreased as area deprivation increased. This result is not different from what has been observed in previous European studies that have investigated the relationship between exposure to green space for women and adverse pregnancy outcomes of low birth weight and preterm birth [19,21]. Indeed, similar results have been observed in a previous general population study from New Zealand; a study of the association between exposure to green spaces and cause-specific mortality in adults from the general population in New Zealand revealed a socioeconomic gradient in exposure to green space [32]. In the aforementioned New Zealand study, one unit increase in deprivation score resulted in a decrease in green space of 11% [32]. 

The findings of the current study suggest that exposure to green space for the entire cohort of women is not beneficial in decreasing the odds of antenatal depression. Additionally, this study could not demonstrate that associations of green space with antenatal depression were stronger for specific population subgroups. 

### 4.2. Comparison of Study Results with Previous Studies

We add to the already existing literature on green space and antenatal depression by exploring the issue of whether green space exposure does or does not reduce the odds of depression during pregnancy. Building on previous research on green space-depression relationships in women from England [13], our New Zealand-based study investigated the green space-depression association for a cohort (*Growing Up in New Zealand*) whilst controlling for confounders, including socioeconomic status, and the length of stay at the current residence. This is the first study in New Zealand that has accounted for self-selection bias for the association between exposure to green space and antenatal depression utilizing the same cohort. The overall relationships in this study were not in the predicted directions. We expected green space to be associated with a lower likelihood of antenatal depression for the entire cohort of women. More importantly, we expected women who were physically active to be less depressed than those who were not physically active. Similarly, we expected women with low levels of education to be less depressed than those with medium or high levels of education. 

McEachan and colleagues utilized the Born in Bradford cohort sample to examine the relationships between exposure and access to green space for women and the odds of antenatal depression [13]. In the Born in Bradford Study, depression amongst the study participants was measured through the administration of the General Health Questionnaires (GHQs). More specifically, a total of four questions were answered on a four-point Likert scale (i.e., 0 to 3) by the study participants. A binary variable, representing the likelihood of depression, was constructed and categorized as “depression” (scores of 0 on all four questions) and “non-depression” (score of 1 on at least 1 question) [13]. In contrast with our study, the Born in Bradford study showed that women residing in the greenest areas had a lower likelihood of antenatal depression when compared to those residing in the least green areas (OR = 0.82 (95% CI = 0.69–0.98)) [13]. Additionally, the association between exposure to green space and antenatal depression in the Born in Bradford cohort was independently identified for women who had attained only low levels of education, as well as women who were physically active throughout their pregnancy. Women with only low levels of education and living in the greenest areas were *less* likely to develop antenatal depression than those living in least green areas (OR = 0.74 (95% CI = 0.59–0.94)) [13]. Those who had achieved the recommended levels of physical activity during pregnancy (at least 150 min of moderate physical activity on average a week [51]) and lived in the greenest areas were *less* likely to develop antenatal depression than those living in the least green areas (OR = 0.63 (95% CI = 0.41–0.97)) [13]. The Born in Bradford study also showed that the relationship between access to green space and antenatal depression was significant for the study participants. Women who lived within 300 m of a major green space were 13% less likely to develop antenatal depression than those who lived >300 m of a major green space [13]. That is, the fully adjusted multivariate regression analyses revealed that women who lived within 300 m of a major green space had lower odds for development of antenatal depression in comparison to those who lived >300 m of a major green space (OR = 0.87 (95% CI = 0.77–0.99)) [13]. No significant green space-depression associations were identified for specific pregnant population subgroups (e.g., those with low levels of education or those who were physically active) while considering access to green space [13]. Some explanations can be given for the contrasting results between our study and Born in Bradford study. One possible explanation is the lack of environmental variation in green space in New Zealand. In New Zealand, cities generally provide a high amount of green space [32,37,52,53]. The high levels of exposure to green spaces due to outdoor vacationing (camping) may be responsible for a lack of variation in exposure to green spaces in New Zealand [27]. Richardson and colleagues went so far as to suggest that, unlike in other places such as in England, green space is not an important determinant of health in New Zealand as green space is everywhere, leading to a lack of variation in exposure [32]. Another explanation for the differences in results between our study and the Born in Bradford study is the inclusion of the neighborhood self-selection variable in the regression analyses. We included the length of stay at the current residence variable as a surrogate for neighborhood self-selection in our regression models whereas the Born in Bradford study did not [13]. However, over the last decade, there has been a housing shortage in Auckland, in particular, due to a failure in the housing market. Therefore, the length of stay at the current residence may not be an ideal surrogate measure of neighborhood self-selection. 

We can also compare our study results to the results of prior general population studies on green space-mental health carried out both within and outside of New Zealand. Our lack of association between exposure to green space and antenatal depression for the entire cohort of women is consistent with the results of a few general population studies on green space and mental health [24,28,54], but not with others [26,27,31,40]. One cross-sectional study assessed the association between exposure to green space and poor mental health in adults from the general population in New Zealand [31]. Poor mental health was judged on the basis of the presence of a short form-36 mental health score in the lowest quartile [31]. This study showed that exposure to green space was associated with lower odds of poor mental health. That is, people residing in the greenest CAUs were *less likely* to report poor mental health than those residing in the least green CAUs (OR = 0.81 (95% CI = 0.66–1.00)) [31]. Another general population study in New Zealand examined the association between exposure/access to green space and mental health amongst adults [27]. In this general population study, mental health was assessed through a number of anxiety/mood disorder treatment counts, and this number included people who had received secondary treatments for mental health disorders, or who had received subsidized prescription drugs for anxiety/mood disorders, or those diagnosed for mental health disorders on the basis of positive laboratory tests for lithium [27]. This study showed that both increases in access to green space (defined as decreased distances to green spaces) and increases in exposure to green spaces in residential environments were associated with decreased anxiety/mood disorder treatment counts. Each decrease in distance to the nearest green space by 100 m was associated with a 3% reduction in anxiety/mood disorder treatment counts [27]. Additionally, an increase in the proportion of greenness in 3 km buffers around the residence by 1% resulted in a decrease in anxiety/mood disorder treatment counts by 4% [27]. An Australian study investigated the association between exposure to green space and mental health amongst adults from the general population [26]. In this green space-mental health study, the participant’s mental health was assessed through the administration of the psychological distress questionnaire which indicated psychological distress [26]. Each questionnaire consisted of 10 questions which were answered on five-point Likert scales (e.g., 1 = none of the time to 5 = all of the time) [26]. Total scores of ≥22 were indicative of the presence of psychological distress [26]. Adults residing in the greenest neighborhoods were *less likely* to develop psychological distress in relation to those residing the least green neighborhoods (OR = 0.83 (95% CI = 0.76–0.92)) [26]. Additionally, people living in the greenest neighborhoods were found to be *less* sedentary compared with people living in the least green neighborhoods (OR = 0.81 (95% CI = 0.77–0.87)) [26]. A recent general population cross-sectional study explored the association between exposure to green space and mental health of residents in the state of Wisconsin, USA [40]. In this study, the mental health of the study participants was accessed through the 42-item depression, anxiety, and stress scale, which indicated symptoms of depression, anxiety, and stress [40]. The results showed that green space was associated with less depression, anxiety, and stress. A 25% increase in the coverage of green space in the residential environment was associated with a decreases in the scores for depression, anxiety, and stress by 1.379, 0.427, and 0.735 points, respectively [40]. A British longitudinal design study conducted in general population adults investigated the association between exposure to green space and mental health disorder of minor psychiatric morbidity [55]. The general health scores obtained from 12-item GHQs were used to assess mental health statuses with higher scores indicating presence of minor psychiatric morbidity [55]. The general health construct within a 12-item GHQ examined a range of questions on mental health issues such as concentration, insomnia, lack of confidence, self-worthiness, happiness, and depression [55]. Analysis of the green space-mental health associations showed that green space exposure was beneficial for women aged >41 years of age resulting in a reduction in the mean GHQ score [55]. The association of green space with minor psychiatric morbidity persisted until old age for women, though beneficial only for exposure to moderate levels of green space, defined as 34–66% green space coverage in the small geographical areas of Wards [55]. In men, the associations of moderate (34–66% green space coverage) and high levels (67–100% green space coverage) of green space with minor psychiatric morbidity were seen in early adulthood appearing at 30 years, and these peak at 41–45 years. These associations remained till 60 years, after which they were not seen in old age [55]. 

A study of the associations between exposure to blue and green spaces (in terms of the visibility of blue and green spaces) and psychological distress in adults in New Zealand failed to establish any connection between exposure to green space and psychological distress [28]. In the aforementioned New Zealand study, investigators assessed the mental health of the study participants through psychological distress scale scores, with higher scores indicating presence of psychological distress [28]. This study showed that blue space visibility was associated with lower scores on psychological distress scales (β = −0.28; *p*-value < 0.001), but green space visibility was not associated with lower scores on psychological distress scales (β = −0.09; *p*-value = 0.455) [28]. This meant that each 10% increase in blue space and green space visibility was associated with a decrease in psychological distress score by 0.28 and 0.09 points, respectively [28]. However, the investigators of this study did not include private gardens in their classification of green spaces, or assess the quality of green spaces, nor control for the length of stay at current residence in the regression analysis [28]. It is possible that their inclusion could have resulted in significant associations between green space visibility and psychological distress [28]. A general population cohort study investigating the association between access to green space qualities and poor mental health has also been carried out through the administration of a 12-item GHQs in Swedish adults. This study showed that access to green space qualities for the entire cohort (OR men = 1.10 (95% CI = 0.7–1.60); OR women = 1.10 (95% CI = 0.80–1.60)) was not associated with the development of poor mental health [54]. However, women who had access to green space and were also physically active were *less* likely to develop poor mental health, in comparison to women without access to green space and who were physically inactive (OR women = 0.30 (95% CI = 0.10–0.90)) [54]. Study limitations included the use of a non-validated measure of physical activity and the use of self-reported measures of access to green space qualities [54]. 

Considering all general population studies, it is possible to say that the directional trends for the associations of green space with mental health outcomes across several studies is essentially mixed. That is, there is a lack of consensus among general population studies conducted within and outside New Zealand on the effect of green space on mental health outcomes. Most general population studies conducted within and outside New Zealand suggest that exposure to green space improves mental health outcomes [26,27,31,40], whilst some others have found no association between exposure to green space and mental health outcomes [28,54]. One possible reason for the lack of consensus between different mental health studies may be variation in the definition of mental health. For example, mental health has been defined in various ways in general population studies, such as poor mental health [31,54], minor psychiatric morbidity [55], psychological distress [26,28], anxiety/stress/depression [24,40], and counts of anxiety/mood disorder [27]. Self-assessment of mental health disorders in most general population studies [24,26,28,31,40,54,55], as opposed to objective assessment of anxiety/mood disorder treatment counts in one general population study [27], is another possible reason for the lack of consensus among the results of general population studies. Nutsford and colleagues stated in their ecological study that objective assessment of mental health status by measuring anxiety/mood disorder treatment count is a better method for assessing mental health status than the subjective measure of anxiety/mood disorder [27].

### 4.3. Strengths and Limitations of This Study

In this study, we unraveled the current set of circumstances regarding the relationship between maternal exposure to green space and the odds of antenatal depression during pregnancy after adjusting for a multitude of confounders. Adjustments were done for important maternal confounders, including age, physical activity, and socioeconomic status. Additionally, we accounted for selection bias by including the variable “length of stay at current residence” in the regression models. Our sample size for the main analyses was comparable to the sample sizes used in previous general population studies (442 [28]; 2479 [40]; 4924 [24]; 7552 [27]; 8157 [31]; 24,945 [54]; 65,407 [55] and 260,061 [26]) and one study on women (7547 [13]) that have investigated similar associations between exposure to green space and depression or other mental health outcomes. Our sample size of 6772 women gave sufficient statistical power in determining the association between exposure to green space and antenatal depression, and to generalize our findings to the rest of the pregnant population of New Zealand.

This study had some limitations. Due to the inherent cross-sectional nature of this investigation, causality could not be determined. The CAUs varied significantly in size over the entire sample. More precisely, the CAUs within the region of the Waikato District Health Board were large in size in comparison with the CAUs within the regions of the Auckland and Manukau District Health Boards. Therefore, exposure misclassification for the estimation of green space for large-sized CAUs could not be ruled out. The possibility of green space exposure misclassification for large-sized CAUs was reduced, at least in part, by performing multilevel analysis. Local Councils and LCDB do not capture data on private gardens. Therefore, we could not include private gardens in classification of green spaces in the current study. While it would have been helpful to investigate the role of access to green spaces by determining distances between home addresses and local green spaces, we did not have the data to do so (e.g., the data on home addresses and the data on road/path networks were not available). Visits to, and time spent in green spaces, have been found to be linked to improvements in mental health outcomes. For example, Magdalena van den Berg and colleagues have demonstrated that visits to and time spent in green spaces are associated with better mental health and vitality scores [56]. In our study, we were not determining whether the participants of the *Growing up in New Zealand* study visited and spend their time in green spaces for any reason (e.g., to establish social connections). Also, we did not have data on the quality characteristics of green spaces (e.g., the safety, esthetics, amenities, and level of maintenance of the area). It has been shown that quality of green space is one factor that determines visits to and subsequent use of green space [57].

### 4.4. Future Directions

Future investigators could involve both objective measurements of access to green spaces by determining distances to local green spaces through the use of roads (people living away from green space often access their local green space through cars) or path networks (people living very close to green space often access their local green space on foot) and the percentage green cover. Private gardens could be included in classification of green spaces. The quality characteristics of the green spaces could also be added as independent variables in the regression models. Gathering information on visits to and time spent in green space is crucial for the determination of the mediators of the association between green space and antenatal depression. 

### 4.5. Comparison between Study Results of Papers Based on the Growing Up in New Zealand Cohort

Two previous pregnant population studies from New Zealand have also utilized the “*Growing Up in New Zealand*” dataset as the data source [33,58]. These studies focused on the associations between: (1) green space and physical activity [33], and (2) green space and pregnancy outcomes of birth weight and gestational age [58]. Despite the lack of association between green space and physical activity, the study of green space and physical activity concluded that exposure to green space could result in better pregnancy health through increased participation in physical activity [33]. The study on green space and pregnancy outcomes of birth weight and gestational age concluded that exposure to green space is not associated with either birth weight or gestational age, based on the cohort as a whole [58]. However, associations for gestational age were found to be significant for specific population subgroups [58]. The current study suggests that exposure to green space is not beneficial for reducing antenatal depression based on either the cohort as a whole, or cohort-specific population subgroups. One reason for the different conclusions could be due to confounding factors, as different sets of independent variables were available for inclusion into the regression models for the different studies. In the study of green space and physical activity, only a few independent variables were used, including age, ethnicity, education, employment status, NZDep2006, and preference for the local lifestyle of the neighborhood [33]. It is possible that some degree of confounding adjustment was not achieved. For example, residual confounding could not have been entirely omitted as we could not control for body mass index due to missing data on body mass index. This meant that green space could have affected physical activity in a positive way, by increasing the odds of participation in physical activity [33]. In contrast, a large range of independent variables were available for the study looking at green space and birth weight and gestational age [58]. These included gestational age, fetus gender, maternal education, employment status, area deprivation, age, self-identified ethnicity, smoking, alcohol consumption during pregnancy, antenatal depression, heart disease or high blood pressure during pregnancy, diabetes mellitus during pregnancy, relationship status with biological father, birth place, parity, lead maternity carer, residential rurality, and time lived in current neighborhood [58]. Similarly, in the current study, a large range of independent variables was available, including maternal age, self-identified ethnicity, relationship status, parity, smoking, alcohol consumption, general health status, physical activity during the first trimester and remainder of pregnancy, education, employment status, residential rurality, area deprivation, and length of stay at current residence. It can therefore be expected that confounding factors were controlled for to a greater extent in the pregnancy outcomes [58] and in the present paper. 

It must be noted that in the investigation of green space and pregnancy outcomes [58] subgroup analyses suggested that exposure to green space was associated with gestational age for women with low levels of education. In contrast, due to the lack of interactions between green space and relevant factors, we did not perform subgroup analysis for the investigations of green space and physical activity [33], and green space and antenatal depression. The interaction analysis may also go some way to explaining the differences in outcomes from the three *Growing Up in New Zealand* studies. 

## 5. Conclusions 

The results of our study suggest that exposure to green space is not an important driver of better mental health (i.e., less antenatal depression) across the cohort of women in our study. Our result should be considered in the context of other studies by policymakers who are involved in policies on the construction of new residential properties in areas of green space. It remains unclear as to whether the provision of new residential areas in greener environments through urban planning could prove beneficial to the mental health of women by lowering odds of antenatal depression and preventing adverse health outcomes associated with antenatal depression. More studies focusing on pregnant women are needed, in both similar and different social contexts, to determine the associations between green space and antenatal depression, considering both exposure and access to green space. 

## Figures and Tables

**Table 1:** Demographics and other characteristics of antenatal mothers and bivariate analyses for antenatal depression used to assess the effect of green space on antenatal depression from the *Growing Up in New Zealand* study cohort.

Variables (*n* = 6772) *	Descriptives *n* (%) or Mean (SD)	Bivariate Analyses Test Statistic (*p*-Value for Chi-Square or F Test)
***Demographics***		
**Age (years), *n* (%)**		
<20	325 (5)	147.31 (<0.001)
20–24	992 (15)	
25–29	1651 (24)	
30–34	2108 (31)	
35–39	1411 (21)	
≥40	285 (4)	
**Education,** ***n*** **(%)**		
No secondary school	485 (7)	123.47 (<0.001)
Secondary school	1610 (24)	
Diploma	2068 (30)	
Bachelor’s degree	1532 (23)	
Higher degree	1058 (16)	
**Employment status, *n* (%)**		
Employed	3636 (54)	93.83 (<0.001)
Unemployed	543 (8)	
Student	455 (7)	
Not in workforce	1822 (27)	
**Self-identified ethnicity,** ***n*** **(%)**		
European	3576 (53)	202.76 (<0.001)
Māori	933 (14)	
Pacific	1001 (15)	
Asian	1002 (15)	
Middle Eastern/Latin-American/African	145 (2)	
Other or New Zealander	96 (1)	
**Relationship status,** ***n*** **(%)**		
No relationship	125 (2)	115.04 (<0.001)
Dating, not cohabiting	278 (4)	
Cohabiting	2312 (34)	
Married or civil union	4038 (60)	
**Parity, *n* (%)**		
First born	2833 (42)	0.68 (0.41)
Subsequent	3932 (58)	
***Health behaviors and health***		
**Smoking,** ***n*** **(%)**		
No	5473 (81)	101.53 (<0.001)
Yes	656 (10)	
**Alcohol consumption,** ***n*** **(%)**		
No drinking during pregnancy	4851 (72)	9.12 (0.003)
Any drinking during pregnancy	1904 (28)	
**Physical activity during the first trimester of pregnancy, *n* (%)**		
No	4243 (63)	2.91 (0.088)
Yes	1890 (28)	
**Physical activity after the first trimester of pregnancy, *n* (%)**		
No	4700 (69)	0.64 (0.42)
Yes	1433 (21)	
**General health status, *n* (%)**		
Poor or fair	698 (10)	168.79 (<0.001)
Good	2306 (34)	
Very good	2382 (35)	
Excellent	1372 (20)	
**Antenatal depression, *n* (%)**		
No	5141 (76)	-
Yes	992 (15)	
***Household characteristics***		
**Area deprivation (NZDep2006),** ***n*** **(%)**		
≤3: low	1684 (25)	90.83 (<0.001)
4–7: medium	2471 (36)	
8–10: high	2615 (39)	
**Length of stay at current residence, mean (SD)**		
Length of stay (years)	4 (6)	1.00 (0.50)
**Residential rurality, *n* (%)**		
Urban	6325 (93)	3.52 (0.06)
Rural	447 (7)	
**District health board region, *n* (%)**		
Auckland	2421 (36)	53.70 (<0.001)
Manukau	2526 (37)	
Waikato	1825 (27)	
***Green space percentage in census area units***		
**Green space percentage,** ***n*** **(%)**		
Low (0–<12%)	1672 (25)	6.87 (0.08)
Medium (12–<21%)	1652 (24)	
High (21–<38%)	1764 (26)	
Very High (38–100%)	1684 (25)	

* The proportion of missing data for each variable is from 0.0 to 9.5%.

**Table 2:** Multilevel analyses for maternal exposure to green space during pregnancy and odds of antenatal depression for the entire cohort used to assess the effect of green space on antenatal depression from the *Growing Up in New Zealand* study cohort.

Association between Maternal Exposure to Green Space and Antenatal Depression (*n* = 6772)				
**Green Space Percentage**	**Model 1 ^a^**	**Model 2 ^b^**	**Model 3 ^c^**	**Model 4 ^d^**
	**OR (95% CI)**	**OR (95% CI)**	**OR (95% CI)**	**OR (95% CI)**
Low	1.00	1.00	1.00	1.00
Medium	1.11 (0.91–1.36)	1.13 (0.92–1.39)	1.12 (0.91–1.37)	1.10 (0.90–1.35)
High	1.12 (0.92–1.36)	1.13 (0.93–1.39)	1.13 (0.92–1.39)	1.12 (0.92–1.38)
Very High	0.95 (0.77–1.17)	1.20 (0.97–1.49)	1.20 (0.96–1.48)	1.22 (0.98–1.51)
**Green space percentage**	**Model 5 ^e^**	**Model 6 ^f^**	**Model 7 ^g^**	
	**OR (95% CI)**	**OR (95% CI)**	**OR (95% CI)**	
Low	1.00	1.00	1.00	
Medium	1.11 (0.90–1.36)	1.10 (0.90–1.36)	1.10 (0.89–1.35)	
High	1.14 (0.93–1.40)	1.14 (0.93–1.40)	1.15 (0.94–1.41)	
Very High	1.22 (0.98–1.51)	1.20 (0.97–1.49)	1.21 (0.96–1.52)	

**^a^** = unadjusted univariate; **^b^** = adjusted for maternal age and self-identified ethnicity; **^c^** = adjusted for maternal age, self-identified ethnicity, smoking, and alcohol consumption during pregnancy; **^d^** = adjusted for maternal age, self-identified ethnicity, smoking, alcohol consumption during pregnancy, pre-pregnancy general health status, physical activity during the first trimester and remainder of pregnancy; **^e^** = adjusted for maternal age, self-identified ethnicity, smoking, alcohol consumption during pregnancy, pre-pregnancy general health status, physical activity during the first trimester and remainder of pregnancy, relationship status, and parity; **^f^** = adjusted for maternal age, self-identified ethnicity, smoking, alcohol consumption during pregnancy, pre-pregnancy general health status, physical activity during the first trimester and remainder of pregnancy, relationship status, parity, education, and employment status; **^g^** = adjusted for maternal age, self-identified ethnicity, relationship status, parity, smoking, alcohol consumption, general health status, physical activity during the first trimester and remainder of pregnancy, education, employment status, residential rurality, area deprivation, and length of stay at current residence.

## Abbreviations

The following abbreviations are used in this manuscript:

ArcGIS	Aeronautical Reconnaissance Coverage Geographic Information System
CAU	Census Area Unit
CI	Confidence Interval
EPDS	Edinburgh Postnatal Depression Scale
GHQ	General Health Questionnaire
LCDB	Land Cover Database
NZDep2006	New Zealand Deprivation Index 2006
OR	Odds Ratio
SD	Standard Deviation
USA	United States of America

## References

[1] World Health Organization Depression. http://www.who.int/topics/depression/en/.

[2] Greenberg P.E., Fournier A.A., Sisitsky T., Pike C.T., Kessler R.C. (2015). The economic burden of adults with major depressive disorder in the United States (2005 and 2010). J. Clin. Psychiatry.

[3] Bromet E., Andrade L.H., Hwang I., Sampson N.A., Alonso J., de Girolamo G., de Graaf R., Demyttenaere K., Hu C., Iwata N. (2011). Cross-national epidemiology of DSM-IV major depressive episode. BioMed Cent. Med..

[4] Andrews G. (2001). Should depression be managed as a chronic disease?. BMJ Br. Med. J..

[5] World Health Organization (2001). Mental Health—A Call for Action by World Health Ministers.

[6] Leigh B., Milgrom J. (2008). Risk factors for antenatal depression, postnatal depression and parenting stress. BioMed Cent. Psychiatry.

[7] Ministry of Health (2012). Healthy Beginnings: Developing Perinatal and Infant Mental Health Services in New Zealand.

[8] Accortt E.E., Cheadle A.C.D., Schetter C.D. (2015). Prenatal depression and adverse birth outcomes: An updated systematic review. Mater. Child Health J..

[9] Goedhart G., Snijders A.C., Hesselink A.E., van Poppel M.N., Bonsel G.J., Vrijkotte T.G.M. (2010). Maternal depressive symptoms in relation to perinatal mortality and morbidity: Results from a large multiethnic cohort study. Psychosom. Med..

[10] Chaaya M., Campbell O.M.R., Kak F.E., Shaar D., Harb H., Kaddour A. (2002). Postpartum depression: prevalence and determinants in Lebanon. Arch. Women Ment. Health.

[11] Milgrom J., Gemmill A.W., Bilszta J.L., Hayes B., Barnett B., Brooks J., Ericksen J., Ellwood D., Buist A. (2008). Antenatal risk factors for postnatal depression: A large prospective study. J. Affect. Disord..

[12] Stewart D.E., Robertson E., Dennis C.L., Grace S.L., Wallington T. (2003). Postpartum Depression: Literature Review of Risk Factors And Interventions.

[13] McEachan R.R.C., Prady S.L., Smith G., Fairley L., Cabieses B., Gidlow C., Wright J., Dadvand P., van Gent D., Nieuwenhuijsen M.J. (2016). The association between green space and depressive symptoms in pregnant women: moderating roles of socioeconomic status and physical activity. J. Epidemiol. Community Health.

[14] United States Environmental Protection Agency What Is Open Space/Green Space?. http://www.epa.gov/region1/eco/uep/openspace.html.

[15] Sanders T., Feng X., Fahey P.P., Lonsdale C., Astell-Burt T. (2015). The influence of neighborhood green space on children’s physical activity and screen time: Findings from the longitudinal study of Australian children. Int. J. Behav. Nutr. Phys. Act..

[16] Cetin M. (2015). Determining the bioclimatic comfort in Kastamonu City. Environ. Monit. Assess..

[17] Cetin M. (2015). Using GIS analysis to assess urban green space in terms of accessibility: Case study in Kutahya. Int. J. Sustain. Dev. World Ecol..

[18] Cetin M., Sevik H. (2016). Evaluating the recreation potential of IlgazMountain National Park in Turkey. Environ. Monit. Assess..

[19] Dadvand P., de Nazelle A., Figueras F., Basagaña X., Su J., Amoly E., Jerrett M., Vrijheid M., Sunyer J., Nieuwenhuijsen M.J. (2012). Green space, health inequality and pregnancy. Environ. Int..

[20] Markevych I., Fuertes E., Tiesler C.M.T., Birk M., Bauer C.-P., Koletzko S., von Berg A., Berdel D., Heinrich J. (2014). Surrounding greenness and birth weight: Results from the GINIplus and LISAplus birth cohorts in Munich. Health Place.

[21] Dadvand P., Wright J., Martinez D., Basagaña X., McEachan R.R.C., Cirach M., Gidlow C.J., de Hoogh K., Gražulevičienė R., Nieuwenhuijsen M.J. (2014). Inequality, green spaces, and pregnant women: Roles of ethnicity and individual and neighborhood socioeconomic status. Environ. Int..

[22] Dadvand P., Sunyer J., Basagaña X., Ballester F., Lertxundi A., Fernández-Somoano A., Estarlich M., García-Esteban R., Mendez M.A., Nieuwenhuijsen M.J. (2012). Surrounding greenness and pregnancy outcomes in four Spanish birth cohorts. Environ. Health Perspect..

[23] Barton J., Pretty J. (2010). What is the Best Dose of nature and green exercise for improving mental health? A multi-study analysis. Environ. Sci. Technol..

[24] Bos E.H., van der Meulen L., Wichers M., Jeronimus B.F. (2016). A Primrose Path? Moderating effects of age and gender in the association between green space and mental health. Int. J. Environ. Res. Public Health.

[25] De Vries S., Verheij R.A., Groenewegen P.P., Spreeuwenberg P. (2003). Natural environments-healthy environments? An exploratory analysis of the relationship between greenspace and health. Environ. Plan. A.

[26] Astell-Burt T., Feng X., Kolt G.S. (2013). Mental health benefits of neighborhood green space are stronger among physically active adults in middle-to-older age: Evidence from 260,061 Australians. Prev. Med..

[27] Nutsford D., Pearson A.L., Kingham S. (2013). An ecological study investigating the association between access to urban green space and mental health. Public Health.

[28] Nutsford D., Pearson A.L., Kingham S., Reitsma F. (2016). Residential exposure to visible blue space (but not green space) associated with lower psychological distress in a capital city. Health Place.

[29] Morton S.M.B., Atatoa Carr P.E., Grant C.C., Robinson E.M., Bandara D.K., Bird A., Ivory V.C., Kingi T.K.R., Liang R., Marks E.J. (2013). Cohort profile: Growing up in New Zealand. Int. J. Epidemiol..

[30] Statistics, New Zealand Geographic Definitions. http://www.stats.govt.nz/Census/about-2006-census/2006-census-definitions-questionnaires/definitions/geographic.aspx.

[31] Richardson E.A., Pearce J., Mitchell R., Kingham S. (2013). Role of physical activity in the relationship between urban green space and health. Public Health.

[32] Richardson E., Pearce J., Mitchell R., Day P., Kingham S. (2010). The association between green space and cause-specific mortality in urban New Zealand: An ecological analysis of green space utility. BioMed Cent. Public Health.

[33] Nichani V., Dirks K., Burns B., Bird A., Morton S., Grant C. (2016). Green space and physical activity in pregnant women: Evidence from the growing up in New Zealand study. J. Phys. Act. Health.

[34] Auckland Council Park Extent. http://aucklandopendata.aucklandcouncil.opendata.arcgis.com/datasets/b73e1d6e2fae4515b517db8c975f85c7_0.

[35] Land Cover Database (LCDB) (2013). The New Zealand Land Cover Database (LCDB).

[36] Salmond C., Crampton P., Atkinson J. (2007). NZDep2006 Index of Deprivation.

[37] Witten K., Hiscock R., Pearce J., Blakely T. (2008). Neighborhood access to open spaces and the physical activity of residents: A national study. Prev. Med..

[38] McCormack G.R., Shiell A. (2011). In search of causality: A systematic review of the relationship between the built environment and physical activity among adults. Int. J. Behav. Nutr. Phys. Act..

[39] Mokhtarian P.L., Cao X. (2008). Examining the impacts of residential self-selection on travel behavior: A focus on methodologies. Transp. Res. Part B Methodol..

[40] Beyer K., Kaltenbach A., Szabo A., Bogar S., Nieto F., Malecki K. (2014). Exposure to neighborhood green space and mental health: Evidence from the survey of the health of Wisconsin. Int. J. Environ. Res. Public Health.

[41] Zhang Y., van Dijk T., Tang J., van den Berg A.E. (2015). Green Space Attachment and Health: A comparative study in two urban neighborhoods. Int. J. Environ. Res. Public Health.

[42] Alcock I., White M.P., Wheeler B.W., Fleming L.E., Depledge M.H. (2014). Longitudinal effects on mental health of moving to greener and less green urban areas. Environ. Sci. Technol..

[43] Morton S., Atatoa Carr P.E., Bandara D., Grant C., Ivory V.C., Kingi T.K.R., Liang R., Perese L.M., Pryor J.E., Reese E. (2010). Growing up in New Zealand. A Longitudinal Study of New Zealand Children and Their Families. Report 1: Before We Are Born.

[44] Cox J.L., Holden M., Sagovsky R. (1987). Detection of postnatal depression development of the 10-item Edinburgh Postnatal Depression Scale. Br. J. Psychiatry.

[45] Matthey S., Barnett B., White T. (2003). The Edinburgh Postnatal Depression Scale. Br. J. Psychiatry.

[46] Waldie K.E., Peterson E.R., D’Souza S., Underwood L., Pryor J.E., Carr P.A., Grant C., Morton S.M.B. (2015). Depression symptoms during pregnancy: Evidence from Growing Up in New Zealand. J. Affect. Disord..

[47] Tsai A.C., Scott J.A., Hung K.J., Zhu J.Q., Matthews L.T., Psaros C., Tomlinson M. (2013). Reliability and Validity of Instruments for Assessing Perinatal Depression in African Settings: Systematic Review and Meta-Analysis. PLoS ONE.

[48] Kheirabadi G.R., Maracy M.R., Akbaripour S., Masaeli N. (2012). Psychometric properties and diagnostic accuracy of the Edinburgh Postnatal Depression Scale in a sample of Iranian women. Iran. J. Med. Sci..

[49] Ekeroma A.J., Ikenasio-Thorpe B., Weeks S., Kokaua J., Puniani K., Stone P., Foliaki S.A. (2012). Validation of the Edinburgh Postnatal Depression Scale (EPDS) as a screening tool for postnatal depression in Samoan and Tongan women living in New Zealand. N. Z. Med. J..

[50] Diez Roux A.V. (2002). A glossary for multilevel analysis. J. Epidemiol. Community Health.

[51] The American College of Obstetricians and Gynecologists (ACOG) (2002). The ACOG committee opinion No. 267: Exercise during pregnancy and the postpartum period. Obstet. Gynecol..

[52] Dekrout A.S., Clarkson B.D., Parsons S. (2014). Temporal and spatial distribution and habitat associations of an urban population of New Zealand long-tailed bats (*Chalinolobus tuberculatus*). N. Z. J. Zool..

[53] Freeman C., Buck O. (2003). Development of an ecological mapping methodology for urban areas in New Zealand. Landsc. Urban Plan..

[54] Annerstedt M., Östergren P.O., Björk J., Grahn P., Skärbäck E., Währborg P. (2012). Green qualities in the neighborhood and mental health—Results from a longitudinal cohort study in Southern Sweden. BioMed Cent. Public Health.

[55] Astell-Burt T., Mitchell R., Hartig T. (2014). The association between green space and mental health varies across the lifecourse. A longitudinal study. J. Epidemiol. Community Health.

[56] Van den Berg M., van Poppel M., van Kamp I., Andrusaityte S., Balseviciene B., Cirach M., Danileviciute A., Ellis N., Hurst G., Masterson D. (2016). Visiting green space is associated with mental health and vitality: A cross-sectional study in four European cities. Health Place.

[57] McCormack G.R., Rock M., Toohey A.M., Hignell D. (2010). Characteristics of urban parks associated with park use and physical activity: A review of qualitative research. Health Place.

[58] Nichani V., Dirks K., Burns B., Bird A., Morton S., Cameron G. (2017). Green space and pregnancy outcomes: Evidence from the growing up in New Zealand study. Health Place.

